# Factors intervening in the childbirth experience: a mixed-methods study

**DOI:** 10.1186/s12884-023-06175-3

**Published:** 2024-01-02

**Authors:** Luciana Braz de Oliveira Paes, Márcia Regina Cangiani Fabbro, Beatriz Rosana Gonçalves de Oliveira Toso, Jamile Claro de Castro Bussadori, Mariana Torreglosa Ruiz, Natália Rejane Salim, Monika Wernet, Aline Oliveira Silveira, Flávia Corrêa Porto de Abreu D Agostini

**Affiliations:** 1 Federal University of Sao Carlos, São Carlos, SP 13565-905 Rod. Washington Luís, s/n, Brazil; 2 Western Paraná State University, Cascavel Campus, Paraná, Brazil; 3 Federal University of Triângulo Mineiro, Uberaba, Brazil; 4 University of Brasília, Brasília, Brazil

**Keywords:** Life-changing events, Mixed-methods studies women, Delivery, Postpartum period

## Abstract

**Objective:**

To analyze the childbirth experience focusing on the intervening factors and on the delivery method.

**Method:**

A sequential and explanatory mixed-methods study guided by the World Health Organization document for positive childbirth experiences. The participants were puerperal women in a maternity teaching hospital from inland São Paulo (Brazil). The first quantitative stage involved descriptive analysis with Poisson regression of 265 answers to the “*Termômetro da Iniciativa Hospital Amigo da Mulher e da Criança*” (“Women- and Baby-Friendly Hospital Initiative Thermometer”) questionnaire. The second stage, qualitative, thematically analyzed the interviews conducted with 44 puerperal women who took part in the first stage. Data integration was by connection.

**The results and discussion:**

The analysis by connection showed that among the factors that restricted the positive experience, C-section was predominant (61.9%), understood as an option due to fear of pain, the treatment modality and previous traumas. Restrictions referring to the presence of a companion (99.6%), not having privacy (83%), disrespectful situations (69.5%), too many touches (56.9%) and the absence of skin-to-skin contact (55%), among others, potentiated fear, loneliness, concern, shame, the perception of disrespect and insecurity with the assistance provided. The promoting factors were as follows: choosing the companion (95.4%) for collaborating in the safety perception, not having infections (83.9%), having continuous team monitoring (82.2%) and pain relief methods (78.9%), which were valued by the women.

**Conclusion:**

The intervening factors that promoted positive experiences were related to clinical and protocol-related issues and to service availability. The restrictive factors were associated with excess interventions, deprivation of rights and of choice, absence of privacy and restriction referring to the presence of a companion. Women with a normal postpartum period felt more insecure and disrespected when compared to those subjected to C-sections, whose choices were considered, although they had lower prevalence of skin-to-skin contact. There is an urgent need to apprehend women's experiences and turn them into actions that guarantee their lives in a safe and respectful way.

## Introduction

Throughout the parturition process, women attribute great value to the following: their ability to give birth physiologically, to be informed [1], to have a companion with them [2], to feel in control of the process and to achieve positive outcomes for themselves and the newborn [1]. Childbirth experiences assessed as positive reveal respectful health care access based on scientific evidence and the provision of emotional support [3–7]. However, the descriptions found indicate care with excessive use or underutilization of interventions, immersed in disrespectful and abusive professional behaviors at the time of delivery [4, 5].

In Brazil, despite the ordinances that establish good practices in delivery and birth care, the routine adoption of interventionist practices is perpetuated, such as amniotomy, lithotomy position, venoclysis with oxytocin use, fasting, restriction to the bed, repeated touches, episiotomy, lying position in delivery and early cutting of the umbilical cord [8, 9]. Therefore, even among women at habitual pregnancy risk, the C-section rate in the country is almost 60%, reaching nearly 90% in private hospitals [7]. This combination contributes to high maternal mortality rates [10] and challenges the country to qualify the care provided to women during the prenatal, delivery and puerperium periods [11].

For the WHO [3], the goal is women-centered care throughout delivery, and studies related to the childbirth experience should seek women's voice and perceptions [12]. Thus, this study aims to deepen the knowledge about the childbirth experience from a mixed design study under the following question: 'Which factors interfere in the childbirth experience and how did women understand them?'. The objective was to analyze the childbirth experience with a focus on the intervening factors and delivery method.

## Method

### Study design

A study of a descriptive nature, with a mixed method and of the sequential explanatory type, that attributes more weight in the quantitative data collection (QUANT) to elaborate the qualitative stage (qual^1^), both stages combined by connection [13]. The “QUANT” stage resorted to a cross-sectional design, and the “qual” stage was characterized as descriptive, according to the Consolidated criteria for reporting qualitative research (COREQ) [14].

The analysis was supported by the constructs of the guiding document entitled “WHO Recommendations on intrapartum care for a positive childbirth experience”, which aims at raising the concept of the care experience as a critical aspect to ensure high quality delivery care and better women-centered outcomes, with a holistic approach based on human rights [3].

### Study locus

The research was carried out in a maternity teaching hospital from inland São Paulo (SP), Brazil, a reference for nineteen municipalities in the micro-region and for high- and medium-complexity care in the Unified Health System (*Sistema Único de Saúde*, SUS). The municipality has 120,691 inhabitants, with a Human Development Index (HDI) of 0.785 according to *Instituto Brasileiro de Geografia e Estatística* [15]. The choice of the locus was for convenience, related to the fact that the main researcher was familiar with the field and access methods. This is an Obstetric Center model where delivery is monitored by the medical team.

### Period

The first stage (QUANT) was carried out from January to June 2021, and the second stage (qual) was carried out between July and September 2021, from one to six months after delivery, a period chosen to enable analysis of the first stage.

### Participants

The study population corresponded to puerperal women who gave birth to their children in this hospital, with a sample comprised of 265 women.

### Selection criteria

In the first stage (QUANT), puerperal women were included during normal postpartum or post C-section in Rooming-In who had gone through the parturition process in the last 24 h in a hospital environment. The following reasons excluded women from this stage: having experienced a miscarriage; giving birth on the way to the hospital; having been transferred from another institution; readmissions; non-emancipated adolescents without the presence of a legal guardian; and postpartum women with hearing, visual or cognitive impairments.

In the second stage (qual), the inclusion criterion was having taken part in the first study stage and being willing to share their childbirth experience. It is noteworthy that in mixed-methods studies of the explanatory sequential type, the qualitative sample should comprise individuals who are in the initial quantitative sample, as the intention is to explore the quantitative results in greater depth [16].

Women who had no Internet access were not included in this stage. In addition, the women who did not participate in the second instrument application phase (post-discharge) after three phone call attempts at different times by the researchers were considered losses. There were only 28 follow-up losses and one due to maternal death, totaling 29 losses. It is noted that there were no refusals to participate in this research in any of the study stages.

### Definition of the sample

The “QUANT” stage participants were 265 puerperal women. Precision was calculated considering a bilateral hypothesis test to estimate a prevalence value, $$\left\{\begin{array}{c}H_0:p=p_0\\H_1:p\neq p_0\end{array}\right.$$ where we have the following equation for the absolute tolerable error of the estimate (error margin) considering a 5% significance level: $\varepsilon =1.96\sqrt{\frac{{p}_{0}\left(1-{p}_{0}\right)}{n}}$. Sample calculation was performed based on the number of births in the last 6 months, which presented a monthly mean of 130 deliveries/month in the institution. The prevalence considered was 38.11% normal deliveries (p_0_), with a 95% confidence level and an estimated absolute error margin of 5.8 percentage points [17]. In order to respect the 6-month period of the sample calculation criterion used in this research and to ensure randomization, 45 interviews were conducted per month, 11 per week. The researcher headed to the hospital and invited all puerperal women that met the inclusion criteria, respecting the number of 11 interviews per week.

The sample for the “qual” stage was obtained from the 236 puerperal women who answered the post discharge questions in the first study stage – quantitative (conducted by phone 10 days after delivery). A nominal list of all 236 women who agreed to take part in the second study phase was prepared, and they subsequently assigned a number. The interviews were conducted by means of a manual draw after enumerating the participants in the order corresponding to the post discharge interviews. From the algorithms selected, 44 women are defined, a number that was reached considering data saturation [18].

### Study variables

The data were initially obtained using the *Termômetro da Iniciativa Hospital Amigo da Mulher e da Criança*(T-IHAMC) [19], using questions 17, 18, 22 to 24, 33 to 35, 37 to 39, 42, 47 to 49, 51, 62, 63 and 65 to 68, referring to the following: dilation upon admission, cervix consistency and effacement upon admission; having a companion during the entire hospitalization; having a companion chosen by the women; type of delivery; eating and drinking throughout labor; venous access in labor; moving, changing positions and/or walking during labor; anesthesia/analgesia to relieve pain during labor; number of touches during hospitalization; pain relief methods during labor; amniotomy; Kristeller maneuver; choice of position for childbirth; directed pushing, puerperal infection; not being left on their own by the professionals; whether they felt safe with the service; the newborn going straight to the woman's lap after birth; going through disrespectful situations; having privacy; and having the parturient woman's choices taken seriously by the maternity hospital professionals, given that these variables respond to the objective of this article.

### Instruments used to collect the information

The instruments used were the T-IHAMC questionnaire applied to the women and a form to collect sociodemographic and obstetric data obtained from the participants' medical records and/or prenatal care booklet.

T-IHAMC is derived from the Maternity Safety Thermometer by the English National Health Service [20]. The original version was translated and transculturally adapted to Brazilian Portuguese in Brazil [21], receiving the name “*Termômetro de Segurança na Maternidade*”. A new instrument was created some years later, including Brazilian women's perspectives, giving rise to T-IHAMC [19].

The T-IHAMC has 69 questions divided into three blocks (admission, hospitalization and post-discharge data) and evaluates the quality of the assistance offered based on the practices adopted by the maternity hospital professionals and the outcomes; it also values the women's experience with regard to the care received, which includes being informed, having the opportunity to choose, receiving respectful care and being heard by the professionals [19]. The number of questions filled in depends on what each woman answers while applying the instrument and on what is recorded in the medical chart, as some questions are specific, for example, delivery method: C-section or vaginal delivery. The questionnaire contains dichotomous and multiple-choice answers, with the possibility for the women to choose all that applies. Description of the answers considered meeting or not meeting the WHO recommendations according to most of the answers.

An open model with a triggering question was used for the “qual” interview, namely, Tell me about your childbirth experience?

### Data collection

Data from the stage (QUANT) referring to admission and hospitalization were collected by the researcher (first author of this manuscript) face-to-face at the hospital, in a private room of the maternity ward, as well as post-discharge data, 10 days after delivery by telephone (postpartum questions). The collection procedure lasted six months.

The interviews in the “qual” stage were carried out remotely due to the COVID-19 pandemic through the Google Meet*®* video call platform, recorded and lasting between 30 and 40 min. The interviews were conducted by the main researcher who was previously trained to carry them out and scheduled according to the participants' availability by telephone contact, sending the access link via WhatsApp®. It is worth noting that the participants had previously signed the consent form in the first study stage.

### Data treatment and analysis

The QUANT stage data were stored in an Excel® spreadsheet and analyzed by means of descriptive statistics of the answers through absolute and percentage frequencies and, subsequently, to estimate the Prevalence Ratio of interest, the Poisson regression model with robust variance was used [22]. A 5% significance level was adopted for all the analyses. All the graphs presented were made with the aid of R software, version 4.0.4, and the analyses were performed in SAS 9.4.

The “qual” stage data analysis was performed by means of the Content Analysis technique in its thematic modality, according to Minayo [23]. The analysis was carried out in three stages: a) pre-analysis, b) exploration of the material, c) treatment of the results obtained and interpretation [23], which imply a fluctuating reading to apprehend ideas, concepts, topics that determine the registration units, the context units, text clippings compatible with the categorization and coding for data recording, inference and interpretation of the results and final analysis [23].

After individually analyzing the quantitative and qualitative approaches, the data were integrated by connection [16]. To organize the analysis and provide new ideas [24], it was decided to use the “joint display” method [25], with quantitative results that presented higher and lower scores connected to the qualitative results.

The study was approved with Certificate of Presentation for Ethical Appraisal (*Certificado de Apresentação para Apreciação Ética*, CAAE) 09359119.8.0000.5430 and opinion number 4.678.206 respecting the national guidelines for research with human beings, namely, National Health Council resolutions No. 466/2012 and No. 560/2016. Both the women and the researchers signed the free and informed consent form.

## Results

The first study stage consisted of 265 postpartum women; the majority had a partner (242; 91.32%), self-declared as white-skinned (135; 50.94%), had nine to eleven years of studies (127; 47.92%) and were Catholics (130; 49.06%). A total of 261 women (98.49%) attended prenatal care with a minimum of two and a maximum of seventeen appointments, and most of the women were classified as habitual-risk pregnancies (211; 79.63%).

**Fig. 1:**
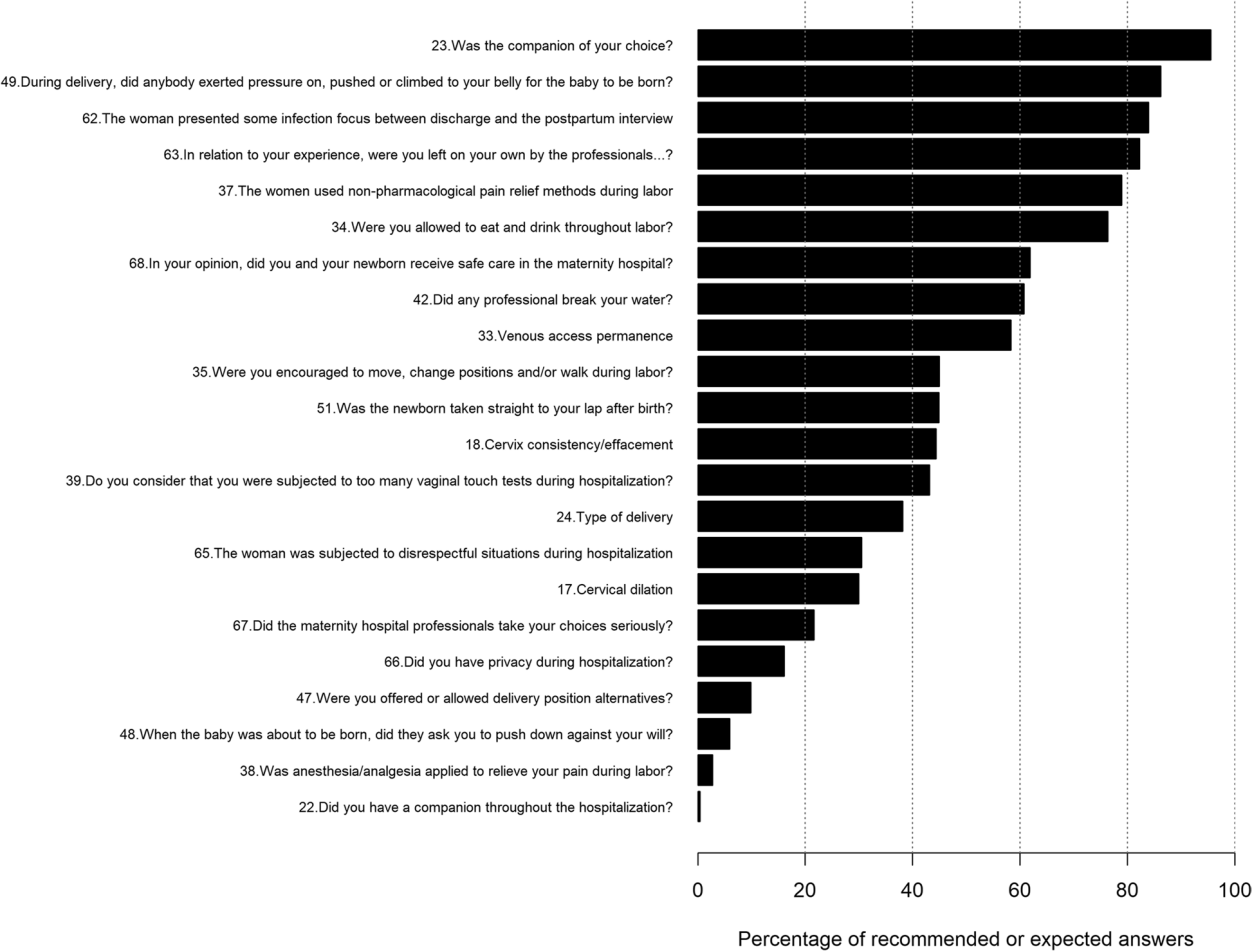
Percentage of answers to the T-IHAMC variables compared to the WHO recommendations

Figure 1 illustrates the variables that most approached the WHO recommendations and those that failed to meet them.

In the figure, it is possible to identify that the women were assisted in terms of the WHO recommendations in the following variables: free choice of a companion (253; 95.47%); being allowed to eat and drink during labor (84; 76.36%); using pain relief methods, such as taking a bath in the shower, staying in the bathtub, sitting on the ball, horse, receiving a massage, using breathing control methods and leaning on the bar, among others (86; 78.9%); nonperformance of amniotomy by a professional from the team (65; 60.75%); not squeezing, pushing or climbing on the stomach to accelerate birth (87; 86.14%); and not having infections between the discharge moment and the post-discharge interview date (198; 83.9%).

In relation to the experience, the majority reported that they received continuous support from the team, even when they were feeling worried, distressed or afraid (194; 82.2%); however, some of the participants did not feel safe with the service they received in the maternity hospital (146; 61.86%).

The questions that presented unrecommended answers were as follows: not having a companion during the entire hospitalization period (264; 99.6%) and the fact that the predominant delivery method was C-section (164; 61.89%) and most of the women were hospitalized without cervical dilation (85; 35.86%), cervix showing hard consistency and no cervix effacement (133; 55.65%).

Other unrecommended practices were venous access permanence (67; 58.26%) and not being encouraged to move, change positions and/or walk during labor (60; 55.05%). In addition, they were not having received anesthesia/analgesia during labor (106; 97.25%) or considering that they were subjected to too many vaginal touch tests during hospitalization (62; 56.88%). Furthermore, not having been offered the possibility to choose the delivery position (91; 90.1%) and reporting directed pushing (95; 94.06%). Finally, after birth, skin-to-skin contact was not allowed (146; 55.09%). During hospitalization: undergoing some disrespectful situation (164; 69.49%); not having privacy (198; 83.9%); and their choices not being taken seriously by the maternity hospital professionals (185; 78.39%).

**Fig. 2:**
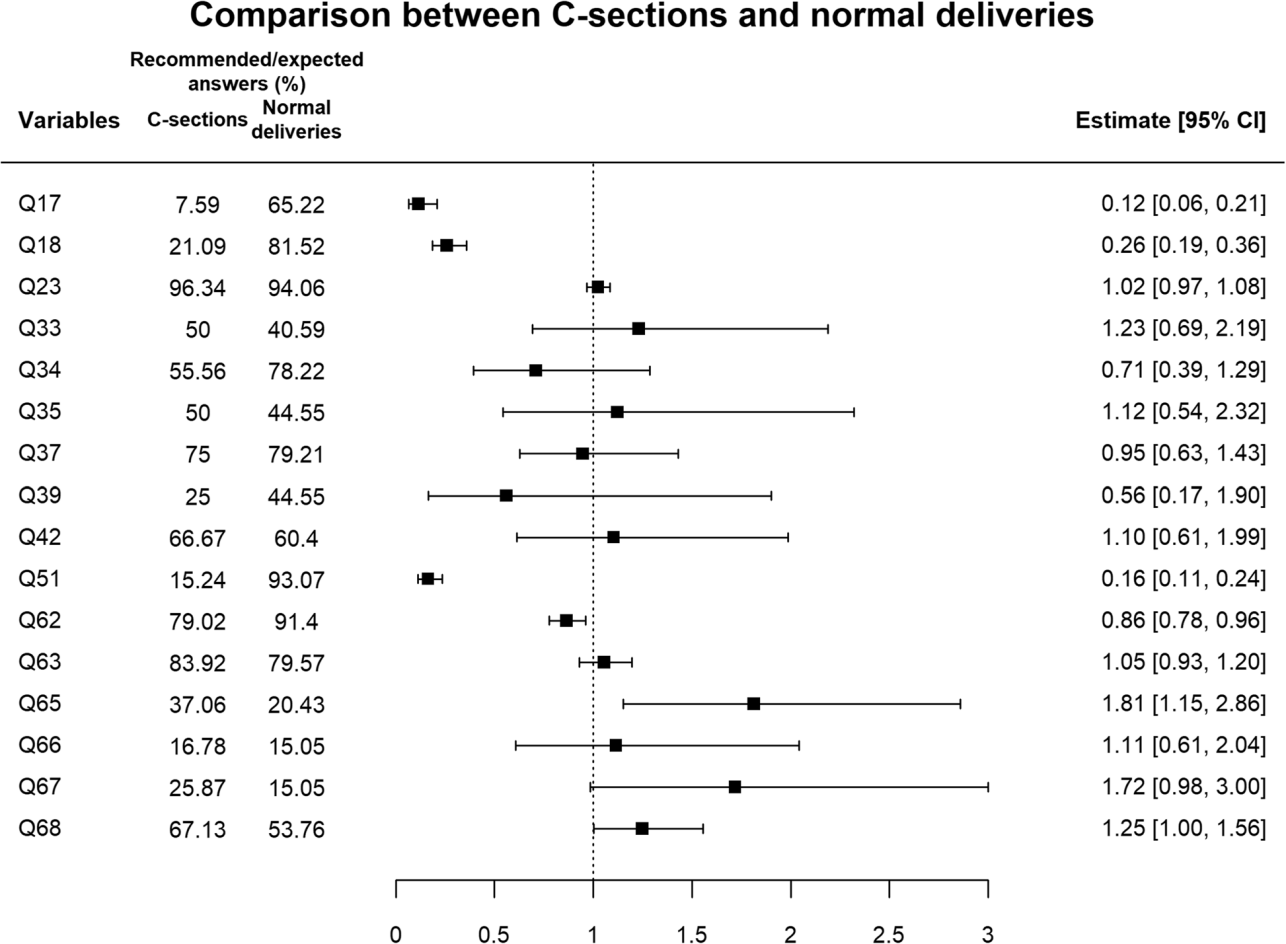
Comparison between the delivery methods and the Prevalence Ratio in meeting or not meeting the WHO recommendations, according to T-IHAMC. Key: 17. Cervical dilation. 18. Cervix consistency/effacement. 23. Was the companion of your choice? 33. Did the venous access remain in your arm during labor? 34. Were you allowed to eat and drink throughout labor? 35. Were you encouraged to move, change positions and/or walk during labor? 37. The woman used non-pharmacological pain relief methods during labor. 39. Do you consider that you were subjected to too many vaginal touch tests during hospitalization? 42. Did any professional break your water? 51. Was the newborn taken straight to your lap after birth? 62. The woman presented some infection focus between discharge and the postpartum interview. 63. In relation to your experience, did the professionals leave you on your own at some moment when you were worried, distressed or afraid? 65. The woman was subjected to disrespectful situations during hospitalization. 66. Did you have privacy during hospitalization? 67. Did the maternity hospital professionals take your choices seriously? 68. In your opinion, did you and your newborn receive safe care in the maternity hospital?

Figure 2 compares the delivery methods and the Prevalence Ratio in meeting the WHO recommendations.

In this figure, it is verified that there was an association between delivery method and questions 17, 18, 51, 62, 65 and 68. In question 17 (dilation at admission), the data show that those who undergo a C-section have an 88% lower prevalence of being hospitalized with a at least 4 cm dilation when compared to patients who had a normal delivery, and in question 18 (Cervix effacement), women who underwent a C-section were hospitalized with a 74% lower prevalence of having their cervix worked on admission.

For question 51, which refers to the newborn going straight to skin-to-skin contact with the mother after birth, the women subjected to C-sections present 84% less prevalence to have the infant in their arms after birth.

The prevalence for infections (question 62) is higher in women subjected to C-sections, as they present 14% lower prevalence to have the recommendation met when compared to those who underwent normal deliveries.

For question 65, the options were disrespectful attitudes that occurred during hospitalization, namely, asking for information about the health of the woman or infant and not obtaining any answer, performing procedures without authorization/permission, feeling disrespected, discriminated against, embarrassed, ashamed or scared, speaking harshly, rudely or shouting at the woman, performing acts such as pinching, pushing, holding hard or hitting, or none of these situations. It was observed that the women subjected to C-sections had 81% more prevalence of meeting the recommendations when compared to those who underwent normal deliveries.

In the safety perception about the care they received at the maternity hospital (question 68), when calculating the Prevalence Ratio, it is identified that the women subjected to C-sections have a 25% greater perception of safety when compared to those who undergo normal delivery.

Forty-four puerperal women were interviewed in the “qual” stage, of which 24 had C-sections and 20 had normal births, aged between 21 and 31 years old (25; 57%), most of them with a partner (41; 93.2%), self-declared as white-skinned (12; 54.54%), 33.58% self-declaring as brown-skinned and 14.72% as black-skin; schooling from nine to eleven years of study (22; 50%), majority of Catholics (19; 43.2%) and Evangelicals (18; 40.9%). All women (44; 100%) attended prenatal care with a minimum of two and a maximum of seventeen appointments, and most of them were classified as habitual-risk pregnancies (211; 79,63%).

From the analysis of the interviews, the coding was done by the phrases and words related to the childbirth experience that were highlighted in the transcribed text, and the corresponding excerpts from the interviews were selected. These phrases or words were regrouped as many times necessary until culminating in two thematic categories. The first one was called “Situations and contexts that restrict positive childbirth experiences” and revealed that the companion-related restrictions potentiated fear, loneliness and concern. Being admitted to the hospital in the labor latent phase, venous access permanence, frequent vaginal touch tests and cardiotocography in the active phase collaborated to the worst experiences. Women opted for C-sections for fear of pain and how they would be treated, lack of information, trauma in a previous delivery, witnessing other women suffering during labor and indications lacking scientific evidence. The institutions have no protocol for C-section indications. Analgesia in vaginal deliveries was denied due to the unavailability of professionals. They reported changing rooms in the expulsion period and not being able to choose the delivery position. The Kristeller maneuver and episiotomy were mentioned in a shoulder dystocia situation in a single case. Directed pushing was understood as bad, as they reported knowing the ideal moment to push. Women subjected to C-sections were separated from their newborns (NBs) in the “Golden Hour” and precluded from breastfeeding during the first hour of life. They felt disrespected by rude treatments and neglect, when pressured to breastfeed, when refusing a medical procedure and by the care being provided by students in the absence of the supervisor. They considered that delivery care was not safe. They felt exposed and embarrassed by the excessive number of students during the procedures and by the presence of men in the rooms, with no separators. A large part mentioned not having the opportunity to choose and, those who had, it was limited to C-section.

The second category is called “situations and contexts that promote a positive childbirth experience” and describes that the best experiences were related to women hospitalized in the active phase of labor and with normal deliveries (NDs), as well as experiencing skin-to-skin contact, breastfeeding in the first hour of life, access to no pharmacological pain relief methods and food during labor. Companions contributed confidence, especially in breastfeeding during the first hour of life and assisting in labor. Women considered it important to receive attention from the team and valued being accompanied by a nurse; those who already had children reported improved care, although they noticed that there were differences between the shifts.

Table 1 presents the integrated analysis resulting from the connection between the quantitative and qualitative results of the intervening factors that restricted positive childbirth experiences and apprehension of this process by the women.

**Table 1:** Integrated analysis chart: Intervening factors that restricted positive childbirth experiences

Variables	QUANT results	qual results
Having companion-related restrictions	264; 99.6%	I6: The companion had to stay in recovery, they put the baby to breastfeed and leave, I was scared, if my husband was there, he would hold me, I felt alone
Not having analgesia in labor	106; 97.25%	I23: I asked for anesthesia, but they said that pain was normal, and there was no anesthesiologist
Asking the woman to push down	95; 94.06%	I1: They encouraged me to push for the baby to be born, but we know the time, it makes you want to I32: They told me to push. It was bad
Not being able to choose the delivery position	91; 90.1%	I29: They took me to the delivery room and showed me a bed; they didn't offer me any other place
Not having privacy	198; 83.9%	I32: I was walking in the hallway in my nightgown, exposed, without rights, in pain. And there are a lot of people at the time of delivery I21: I was ashamed of the companions. In the SUS, if it weren't for that, it would be better than the private one
Choices not taken seriously	Women subjected to C-sections have 81% more prevalence of having their choices taken seriously in relation to those that enter labor	I29: In the SUS we have to accept how things work because everything is already a routine, we're not free to talk, if we talk, they don't like it I37: I was only able to choose the C-section, not the rest
Undergoing some disrespectful situation	1. Those subjected to C-sections have 81% more prevalence of not being disrespected than those who underwent NDs 2. Those who entered labor have 45% fewer prevalence of not being disrespected when compared to those subjected to C-sections	I36: I felt pressured to breastfeed, I felt disrespected I27: I refused oxytocin, then the doctor said: “So you stay there suffering, there are people who are really stupid” I30: They don't respect my opinion, we need to be heard, it was obstetric violence I26: It's not bad for the baby to be born with ND, but the way labor is monitored, for example, the touches I43: I was disrespected because I didn't know what they were doing. The doctor in chief came in quarreling with them and I was crying my pain out
C-section predominance	1. Those subjected to C-sections have 88% less prevalence to be hospitalized with at least 4 cm dilation when compared to those with NDs 2. 74% lower prevalence of having the cervix worked on admission	I4: I went to the hospital with 3 cm dilation but no contractions, but I was in pain, I ended up undergoing a C-section I7: I wanted ND but I saw women crying and shouting in the hospital and got scared, I desisted I8: On the other I suffered a lot in labor, it was a bad experience, I was afraid of the pain, how it would be treated, how it would happen, the touches, everything I15: The private doctor told me to do a C-section I22: I have no information about delivery, the C-section solves it faster, right?
Venous access permanence during labor	67; 58.26%	I18: I had a vein stuck in case I needed a drip
Too many vaginal touch tests	62; 56.88%	I32: Too many touches, they were always by more than one person, there has to be some limit
Movement restrictions during labor	60; 55.05%	I28: What I didn't think good was to do cardiotocography, the contractions were already strong, I wanted to move
Not allowing the newborn to go straight to the mother's lap	146; 55.09% Women subjected to C-sections have 84% lower prevalence of having their newborns on their lap after birth	I44: I think that a woman who undergoes a C-section, the baby could stay with the mother in the recovery period, she wouldn't be worried

Table 2 below presents the quantitative results connected to the qualitative results of the intervening factors that promoted positive childbirth experiences and apprehension of this process by women.

**Table 2:** Integrated analysis chart: Intervention factors that promoted positive childbirth experiences

Variables	QUANT results	qual results
Having the companion of choice	253; 95.47%	I11: At that moment I was not in myself, in “Deliveryland”, my husband tells me some things and he knew my desire I3: I felt safe, protected, knowing that he's involved and sharing the responsibility of having a child
Not having infections	198; 83.9% It is estimated that the women subjected to C-sections have 14% lower prevalence of not presenting infections	I14: I've had no problems so far, no infection in the stitches, everything fine
Not being left on their own by the professionals	194; 82.2%	I40: I had a nurse who helped me, took me to the bathroom to take a shower, I stayed on the ball, she was with me, so she helped me a lot, she massaged me. I believe that having the nurse on my side was the best experience I12: Things got better, that was that humanized delivery. Care evolves very well, with my other son, 13 years ago, they told me “next year you'll be here again” I11: It was two shifts that assisted me, the first team was more attentive, better
Using pain relief methods during labor	86; 78.9%	I12: I was under the shower, they told me to move my hips, dance, move my hips, go on the ball, they massaged my back
Being allowed to eat and drink during labor	84; 76.36%	I18: I was allowed to eat, drink, walk, I also had a massage, they told me to go to the shower
Seeking the hospital already in labor	164; 61.89%	I28: I went to the hospital already in labor, this one was faster than the others, when I got there I was already with 7 cm dilation and it was born quickly

Only in the quantitative stage was safety considered a positive experience, whereas the women reported not feeling safe in the qualitative stage. In addition, when calculating the Prevalence Ratio, it was possible to identify a greater safety perception in the women who underwent C-sections, as shown in Table 3.

**Table 3:** Integrated analysis chart: Contradictory intervening factors in the qualitative and quantitative data connection for positive childbirth experiences

Variables	QUANT results	qual results
Feeling safe with the care received in the maternity hospital	146; 61.86% 1. The women subjected to C-sections had 25% better safety perceptions when compared to those who entered labor 2. The women who entered labor had 25% lower prevalence of feeling safe when compared to those subjected to C-sections	The qualitative results diverge from the quantitative ones I12: The doctors did not stay with me sometimes, they were talking or quarreling among them, then I thought: “Are they really paying attention to me?” I26: I didn't feel safe, my child was born with no pediatrician nearby I41: It wasn't safe, the student didn't know what to do, I grabbed my son, otherwise he'd fall to the ground. You need to have the head professional there I17: I did prefer the C-section, I felt safer

## Discussion

The results of the current study show some interaction between factors that promote or restrict the outcome of a positive childbirth experience.

Among the restrictive factors, the issues related to guaranteeing rights (having information, having a companion, anesthesia/analgesia and freedom to choose the delivery position) were evidenced. The study pointed out practices such as lack of privacy at the time of delivery or maintaining venous access, even if without indication. The results showed practices that represent violations of rights, such as excessive number of vaginal touches for the students' objective training in the context of an institution linked to teaching. Perpetuation of obsolete and harmful practices according to diverse consolidated scientific evidence, such as directed pushing and movement deprivation during labor, intersected each other and contributed to this scenario.

When analyzing and discriminating these factors based on the women's perspective, the results indicate the need for a change in the obstetric practices in order to achieve positive childbirth experiences. Some findings of this study, such the predominance of C-sections, disrespectful situations or denying the possibility to choose triggered negative delivery and childbirth experiences, in opposition to the national and international recommendations for a positive experience [3, 26, 27]. This fact corroborates with the WHO guideline which advocates that the lower the intrapartum care quality, the more impaired the global standards for the promotion of women-centered assistance will be [28].

In Brazil, it was evidenced that the implementation of good care practices during labor and delivery is greater than the reduction in the number of obstetric interventions that are not recommended in routine care, as it is easier to introduce new care processes than to withdraw old consolidated practices [29]. However, an obstetric assistance model that favors fear and a feeling of loneliness still prevails in a context marked by the invisibility of parturient women's rights. This model will never favor positive experiences, as it is recurrently linked to maternal-neonatal harms and mortality [3].

Thus, this study adds to previous criticisms about the inadequacy of institutions linked to health education in terms of transforming people's bodies and to a history turning people into practical training objects. The female body is objectivized, and women's right to privacy and autonomy is annihilated in the name of the ‘school’, of the ‘belly or vagina school’, paraphrasing Diniz [30]. Changes are urgently needed because, when indulging in these behaviors, training institutions reiterate and naturalize disrespect for women, and we can say the hierarchical relationship between professionals and health care users. The literature about humane and fair care constantly points to this issue, and it continues to be perpetuated. Diniz et al [30] . exemplify that, in practice, future professionals are taught that patients do not have the right to informedly choose or refuse and that the teaching needs of those trained are more important than the parturients' autonomy or bodily integrity.

When taking the delivery method, the women who had normal deliveries went through more disrespectful situations when compared to those subjected to C-sections. In the qualitative stage, these women reported rude manners and neglect when forced to breastfeed or when they refused a medical action, which generated feelings of trauma, fear, suffering and a death sensation. Worldwide, including Brazil, a large number of parturients experience disrespectful and abusive behaviors, which infringes women's right to receive respectful care [31–34]. The consequences of these abuses are reflected in women's health, both physical and mental [34–36], and, in some cases, with repercussions for the newborns [35], which reflects in an increase in the number of maternal “near miss” and maternal–fetal mortality situations [37]. Likewise, it can be assumed that experiencing obstetric violence will change the choice regarding the delivery method to C-section in subsequent pregnancies, both in Brazil and in other countries [35, 38–40].

The associations showed that women subjected to C-sections were hospitalized with fewer chances of having a dilated and effaced cervix, contrary to the WHO recommendation regarding hospitalization in the active phase of labor [3]. In contrast, being admitted to the hospital already in labor notoriously collaborated with positive experiences, understood by the lower number of procedures and interventions. Globally, unnecessary C-section rates have increased progressively in recent decades, representing 21.1% of all live births [38]. Latin American countries have the highest C-section rates, with 44.3% of births, and specifically, Brazil has the second highest C-section rate in the world [38], reaching 57.2% of all births in 2020 [41]. It is also worth mentioning that the results of this research showed that the women subjected to C-sections are more likely to having postpartum infections, which, at the global level, are one of the causes for the increase in maternal morbidity and mortality, dissatisfaction in the patients, longer hospitalization times and higher treatment costs [42, 43]. In addition to that, postpartum infection is the third leading cause of maternal death in Brazil [44].

Added to the C-section scenario, in the current research the women and newborns were deprived of skin-to-skin contact, mainly among women subjected to C-sections. Deprivation of this practice goes against the best scientific evidence that advocates the Golden Minute/Hour, thus generating concern and insecurity in the parturient women. Corroborating this research, a study reports interventions in healthy newborns that were deprived of skin-to-skin contact, with the persisting need for evidence-based practices [45] with the involvement of health professionals for the concrete practice of skin-to-skin contact, through continuing education and the creation of protocols [46]. This is because mother–child deprivation and separation constitute neonatal violence and have direct repercussions on the establishment of bonding and secure attachment, as well as on health, with lifelong effects on both [47].

On the other hand, the integrated analysis showed positive impacts on the childbirth experience, such as the guarantee to choose a companion, which exerts an effect of women's safety and support perceptions. This corroborates another study which showed that, in addition to conveying safety, the presence of a companion is associated with various beneficial practices and with lower risks of being victims of obstetric violence [48].

Care availability can be an essential point for changing the scenario of traumatic childbirth [49], increasing women's satisfaction and restoring confidence in maternity hospital professionals [50]. In this scenario, obstetric nurses and midwives can favor the physiological development of labor, enhancing the use of beneficial practices, in addition to providing assistance focused on women's role [5, 51, 52]. In addition, if the deficit in the number of duly trained midwives was eliminated, two-thirds of the maternal and neonatal deaths might be prevented, saving more than 4.3 million lives per year until 2035 [53].

The predominance of some intrapartum recommendations, such as not using the Kristeller maneuver and amniotomy, the use of pain relief methods during labor and being allowed to eat and drink during labor and delivery, contributed good experiences in the current research, which was also observed in a national study regarding the Evaluation of the *Rede Cegonha*, with improvement of these indicators from 2012 to 2017 in all regions of the country [29].

The improvement in care compared to previous births has been reported as a moment of transition from the national reality [29] and showed reductions in the inequalities in childbirth care and in the number of interventions. However, the current study also shows that there is a difference between shifts, corroborating studies that cite barriers in the implementation of protocols in childbirth care in Brazil [49].

Most of the women felt safe with the care they received; however, when associated with the type of delivery, it was possible to verify that the women subjected to C-sections have a greater safety perception when compared to those who undergo normal deliveries, showing that these latter perceive absence or inadequate assistance by the professionals during childbirth, feel alone with students and without supervision from the professor when listening to the physicians' conversations and quarrels. This panorama refers to apprehension of the structural and organizational aspects of the services and the work processes in the assistance provided to women during labor and birth, given that they interfere in the identification of causes and timely management of the prevention of maternal and neonatal death [54].

This study had limitations, such as the fact that the data were collected at a vulnerable time for the women who were recovering from childbirth, in addition to being conducted in a single maternity hospital. The COVID-19 pandemic imposed obstacles, especially when the interviews were conducted via video calls. The T-IHAMC tool was unable to cover all the singularities experienced by the women, an aspect that is difficult to quantify; in addition, the absence of a score for this thermometer posed challenges to the statistical analysis, an obstacle to data integration.

On the other hand, using both approaches helped overcome possible limits, as the shortcomings of one method were able to be compensated by the potential of the other and, thus, a global perspective is contributed by using the mixed method.

## Conclusion

The intervening factors that promoted positive experiences were related to clinical and protocol-related factors or to service availability. The restrictive factors were related to excessive interventions, deprivation of rights and choice, non-recognition and denial of women's autonomy, insecurity, lack of privacy and restriction referring to the presence of a companion. The association regarding the type of delivery showed that the women subjected to C-sections felt safer and had fewer chances of being disrespected.

This research also denounces the cruel setting where women undergo their childbirth experiences, showing that they do not have their rights assured at the time of delivery. Some issues have been a reality for centuries and remain in the birth scenario; however, this fact cannot represent conformity, requiring urgent measures to enable compliance with so many recommendations and policies already in force, that is, the WHO recommendations were not followed in all situations.

Scientific evidence needs to be used as a reference for transforming woman-centered care, in addition to advances in theoretical frameworks that include valuing cultural and personal experiences. In addition, new studies are suggested addressing the impacts of traumatic deliveries on the mental health of women and newborns alike.

It is hoped that this study will collaborate in the elaboration of policies, protocols and norms, which will ensure that all women and their children not only survive but also have access to the best, dignified and respectful care and experience this moment with intensity and dignity.

## Data Availability

The quantitative datasets used and/or analyzed during the current study are available according to the Materials and Methods section. The full transcribed interviews, code and metadata about qualitative studies are not publicly available due to their qualitative nature but are available from the corresponding author upon request.

## Abbreviations

QUANT: Quantitative
qual: Qualitative
WHO: World Health Organization
T-IHAMC: Termômetro da Iniciativa Hospital Amigo da Mulher e da Criança
NB: Newborn
ND: Normal delivery

## Footnotes

1 In this type of study, the quantitative stage is identified with upper-case letters and the qualitative one with lower-case letters.
